# Electromyographic, cerebral, and muscle hemodynamic responses during intermittent, isometric contractions of the biceps brachii at three submaximal intensities

**DOI:** 10.3389/fphys.2014.00190

**Published:** 2014-06-11

**Authors:** Yagesh Bhambhani, Jui-Lin Fan, Nicolas Place, Javier Rodriguez-Falces, Bengt Kayser

**Affiliations:** ^1^Department of Occupational Therapy, Faculty of Rehabilitation Medicine, University of AlbertaEdmonton, AB, Canada; ^2^Institute of Sports Sciences and Department of Physiology, Faculty of Biology and Medicine, University of LausanneLausanne, Switzerland; ^3^Lemanic Neuroscience Doctoral School, University of LausanneLausanne, Switzerland; ^4^Department of Electrical and Electronic Engineering, Public University of NavarraPamplona, Spain

**Keywords:** biceps force, electromyography, cerebral blood flow and oxygenation, muscle blood flow and oxygenation

## Abstract

This study examined the electromyographic, cerebral and muscle hemodynamic responses during intermittent isometric contractions of biceps brachii at 20, 40, and 60% of maximal voluntary contraction (MVC). Eleven volunteers completed 2 min of intermittent isometric contractions (12/min) at an elbow angle of 90° interspersed with 3 min rest between intensities in systematic order. Surface electromyography (EMG) was recorded from the right biceps brachii and near infrared spectroscopy (NIRS) was used to simultaneously measure left prefrontal and right biceps brachii oxyhemoglobin (HbO_2_), deoxyhemoglobin (HHb), and total hemoglobin (Hb_tot_). Transcranial Doppler ultrasound was used to measure middle cerebral artery velocity (MCAv) bilaterally. Finger photoplethysmography was used to record beat-to-beat blood pressure and heart rate. EMG increased with force output from 20 to 60% MVC (*P* < 0.05). Cerebral HbO_2_ and Hb_tot_ increased while HHb decreased during contractions with differences observed between 60% vs. 40% and 20% MVC (*P* < 0.05). Muscle HbO_2_ decreased while HHb increased during contractions with differences being observed among intensities (*P* < 0.05). Muscle Hb_tot_ increased from rest at 20% MVC (*P* < 0.05), while no further change was observed at 40 and 60% MVC (*P* > 0.05). MCAv increased from rest to exercise but was not different among intensities (*P* > 0.05). Force output correlated with the root mean square EMG and changes in muscle HbO_2_ (*P* < 0.05), but not changes in cerebral HbO_2_ (*P* > 0.05) at all three intensities. Force output declined by 8% from the 1st to the 24th contraction only at 60% MVC and was accompanied by systematic increases in RMS, cerebral HbO_2_ and Hb_tot_ with a leveling off in muscle HbO_2_ and Hb_tot_. These changes were independent of alterations in mean arterial pressure. Since cerebral blood flow and oxygenation were elevated at 60% MVC, we attribute the development of fatigue to reduced muscle oxygen availability rather than impaired central neuronal activation.

## Introduction

Despite the importance of upper limb use in our day-to-day life, particularly during occupational and recreational activities, the underpinning neuromuscular and hemodynamic changes during upper limb muscle contractions at light, moderate, and high intensities have not been well documented. Voluntary muscular force results from cortical neuronal activation leading to motor unit recruitment at the spinal level. Depending on the type (e.g., static or dynamic), intensity, and duration of muscle contraction, the force exerted may be limited by mechanisms at any level, from the cortical neuronal activation to the interaction of contractile proteins in the muscle fibers (Gandevia, 2001; Enoka and Duchateau, 2008). If the contractions are sufficiently intense and/or prolonged, muscle fatigue, defined as a transient loss of force generating capacity, develops (Gandevia, 2001; Enoka and Duchateau, 2008). Classically, muscle fatigue is described as central, when the force generating capacity is impaired through mechanisms proximal to the neuromuscular junction, and peripheral, when distal from the junction (Gandevia, 2001; Enoka and Duchateau, 2008). The extent of central and peripheral fatigue and their interplay depend on the type of muscular activity and the particular circumstances. Surface electromyography (EMG), combined with transcutaneous electrical stimulation, has been extensively used to evaluate the relative contributions of central and peripheral factors limiting muscle force development (Burnley et al., 1985; Gauche et al., 2009; Millet et al., 2011; Neyroud et al., 2013). But despite decades of research, the understanding of the underlying mechanisms remains incomplete.

Near infrared spectroscopy (NIRS) allows simultaneous examination of cerebral and muscular hemodynamic responses [i.e., changes in oxyhemoglobin (HbO_2_) and deoxyhemoglobin (HHb)] (Ainslie et al., 2007; Bhambhani, 2012). Several studies have indicated that cerebral tissue desaturation might be a performance-limiting factor during continuous (Gonzalez-Alonso et al., 2004; Bhambhani et al., 2007; Subudhi et al., 2009; Billaut et al., 2010; Rasmussen et al., 2010) and intermittent (Shibuya et al., 2004a,b) high intensity dynamic exercise involving a large muscle volume (such as running or cycling). It was hypothesized that the reductions in cerebral blood flow, cerebral HbO_2_, and total hemoglobin (Hb_tot_), associated to the hyperventilation-induced hypocapnia during heavy exercise may be involved in the disengagement from the effort at the point of exhaustion (Amann et al., 2007; Amann and Kayser, 2009; Subudhi et al., 2009; Rasmussen et al., 2010). In contrast, no cerebral tissue desaturation is observed during exercise involving a smaller muscle volume, such as during unilateral static (Pereira et al., 2007) or dynamic (Matsuura et al., 2011; Gomes et al., 2013) knee extensions. Since both HbO_2_ and Hb_tot_ are compromised at the muscle (Matsuura et al., 2011; Gomes et al., 2013), it is thought that fatigue of the lower limbs during moderate to high resistance is peripherally mediated, in part by limited muscle blood flow and oxygen availability rather than compromised motor drive (Place et al., 2009). In agreement, observations during intermittent isometric knee extensions indicate the presence of a critical intensity threshold, above which fatigue is primarily due to metabolic disturbances in the exercising muscle (Burnley et al., 1985). However, the limiting role of peripheral fatigue for small volume muscle efforts has recently been questioned. Neyroud et al. (2012) found that electrical stimulation at the point of task failure elicited the target force (20% MVC) during sustained isometric knee extension, which indicates that the lack of force generating capacity associated with peripheral impairment was not responsible for limitation the duration of the task. Instead, they concluded that task failure from a low intensity, sustained isometric contraction was mainly due to central/motivational factors, whereas the MVC force loss was largely explained by the contractile failure of the muscle. Another study by the same group (Neyroud et al., 2013) found that time to task failure during a sustained isometric contraction at 50% of MVC with the biceps brachii was shorter than that performed with plantar flexors or thumb adductor. While no cerebral or muscle hemodynamic measurements were performed, the researchers speculated that reduced muscle oxygen availability due to restricted blood flow was likely implicated.

To complement these findings we combined surface EMG with cerebral and muscle NIRS to evaluate the neuromuscular and hemodynamic responses during 24 intermittent isometric contractions of the biceps brachii at three submaximal intensities: 20, 40, and 60% MVC. These intensities were selected on the basis of previous studies (Felici et al., 2009; Muthalib et al., 2010), and our pilot data, which suggested that fatigue development could be evidenced at 60% MVC during this protocol. Research (Maton and Gamet, 1989; Fallentin et al., 1993) has demonstrated that during sustained submaximal contractions up to 80% MVC, a gradual rise in EMG amplitude normally occurs. This rise in EMG activity is attributed to the recruitment of additional motor units and increased firing rate necessary to counteract the increasing fatigue. Therefore, we hypothesized that during these biceps brachii contractions at 20, 40, and 60% MVC: (1) the root mean square of the EMG (RMS), and the cerebral and muscle hemodynamic responses would change disproportionately to the force output at 60% MVC, (2) the force output would correlate better with the changes in muscle rather than cerebral hemodynamic responses, and (3) the changes in RMS and cerebral HbO_2_ would be significantly correlated.

## Materials and methods

### Subjects

Eleven healthy subjects (nine males and two females) with a mean age of 34.5 ± 5.2 years (mean ± *SD*) and body mass index of 24.2 ± 2.4 kg.m^−2^ participated. The local research ethics committee approved the study protocol. The subjects were informed regarding the procedures, and signed consent was given prior to participation. All subjects were recreationally active but none of them were involved in systematic resistance training programs during the course of the study.

### Test protocol

Each subject completed a single testing session using a custom built ergometer equipped with an adjustable handle and strain gauge as previously described (Neyroud et al., 2013). After a short warm-up consisting of 8–10 submaximal isometric contractions at approximately 50% of estimated MVC, the subject performed three MVCs by exerting maximal force with the right forearm at an elbow angle of 90°. Contractions were interspersed with 30 s rest to facilitate sufficient recovery. The highest of the three force values was retained as MVC force. The subjects then did 24 intermittent isometric contractions of the biceps brachii at 20, 40, and 60% MVC in systematic order, at 12 contractions per minute (2.5 s contraction and 2.5 s relaxation) in rhythm with a metronome. The average force output of the 24 contractions was expressed as a percentage of MVC. Pilot testing indicated that a 3-min rest interval between the three intensities was sufficient to minimize the effects of fatigue and allow the cerebral and muscle hemodynamic responses to recover to baseline values. Magnetic resonance spectroscopy findings indicate near maximal resynthesis of intramuscular phosphocreatine and ATP occurs after 3 min of rest following high intensity exercise (Prompers et al., 2014). The subjects were instructed to exhale during the contractions and inhale during relaxation so as to avoid any Valsalva maneuvers at the higher intensities, which could influence the cerebral hemodynamic measurements (Pott et al., 2003).

### Physiological measurements

All signals were acquired using an analog-to-digital converter (PowerLab 16/35, ADInstruments, Australia) with commercially available software (LabChart, version 7.2, ADInstruments, Australia), and stored on disk for subsequent analysis.

#### Biceps brachii performance

Force output of the elbow flexors was recorded at 2 kHz using a custom designed ergometer with the right forearm in the vertical and supinated position and the elbow flexed at 90°. The seat height was adjusted so that the shoulder was in line with the base of the ergometer, which was mounted on a tabletop. The subject exerted force against the ergometer handle, which was fitted with a strain gauge (SAS 200 kg, sensitivity 1.998 mV/V, SWJ, China). The ergometer was interfaced with the AD-system (PowerLab 16/35, ADInstruments, Australia) so that the desired force output at the three intensities could be displayed on a computer monitor for visual feedback to the subject.

#### Electromyography recordings

The EMG activity of the biceps brachii muscle was recorded with pairs of silver chloride (Ag/AgCl) circular (recording diameter of 1 cm) surface electrodes (Kendall Meditrace 100, Tyco, Canada) positioned lengthwise over the middle of the muscle belly with an inter-electrode (center-to-center) distance of 2 cm. The reference electrode was placed over the ipsilateral wrist. The electrode site was shaved lightly abrading the skin and cleaning with alcohol to minimize electrical resistance (verified <10 kΩ). The myoelectrical signals were amplified with a gain of 1000 and filtered using a bandwidth with frequency between 10 and 500 Hz (LabChart version 7.2, ADInstruments, Australia). The filtered EMG signals were sampled at 2 kHz by the analog-to-digital converter. A common mode rejection ratio of 90 dB was used to supress extraneous noise and enhance the signal to noise ratio. For each MVC, the RMS was quantified during the period (approximately 0.5 s) where the peak force was developed. For the submaximal contractions at each intensity, the RMS was quantified during the force plateau (approximately 1 s) observed during individual contractions.

#### Cerebrovascular and cardiovascular measurements

Bilateral middle cerebral artery velocities (MCAv, as an index of cerebral blood flow, CBF) were measured using a 2-MHz pulsed Doppler ultrasound system (ST3 Spencer technology, USA). The probes were positioned over the temporal windows and firmly held in place with an adjustable headband. The signals were recorded at depths ranging from 43 to 54 mm. The bilateral MCAv were averaged to represent global CBF during rest and exercise. Beat-to-beat systolic (SBP), diastolic (DBP), and mean arterial blood pressure (MAP) was monitored with finger photoplethysmography (Finometer Midi, Finapress Medical Systems, Netherlands) using standardized procedures (Ainslie et al., 2007).

#### Cerebral and muscle hemodynamic using NIRS

Cerebral and muscle HbO_2_ and HHb were recorded simultaneously during the baseline, exercise and recovery phases using continuous dual-wavelength NIRS (Oxymon, Artinis Medical Systems, Netherlands). The cerebral probe was modified to fit on the adjustable headband used to record the Doppler blood flow measurements (see above). The probe was placed on the left prefrontal lobe approximately 3 cm from the midline of the forehead, just above the supraorbital ridge (Bhambhani et al., 2006; Rasmussen et al., 2007). The muscle probe was placed adjacent to the EMG electrodes over the belly of the right biceps brachii to ensure metabolic homogeneity (Pappas et al., 2002; Felici et al., 2009). It was secured with a dark tensor bandage to minimize artifact from stray light. Prior to each test, the cerebral and muscle NIRS probes were calibrated according to the manufacturer's instructions. All signals were recorded at 50 Hz and stored for subsequent analysis. The sum of HbO_2_ and HHb was used to calculate Hb_tot_, which was considered an index of localized blood flow in cerebral and muscle tissue (Boushel et al., 2000). The difference between HbO_2_ and HHb (Hb_diff_) was used as an index of tissue oxygen extraction. Delta values of these NIRS variables were calculated as follows: peak value attained at each exercise intensity minus the baseline value prior to the onset of exercise. The baseline was the average 20-s value between 2:30 to 2:50 of the 3 min resting period prior to the onset of each exercise intensity. The final 10 s were not used in calculating the baseline value because anticipation of exercise can alter the hemodynamic responses (Colier et al., 1995).

### Statistical analysis

Normality of the data was initially verified using the Shapiro-Wilk test. Thereafter, One-Way repeated measures analysis of variance (ANOVA) was used to examine the differences in the group mean values of the physiological responses at 20, 40, and 60% MVC. Two-Way repeated measures analysis of covariance (ANCOVA) was used to examine the changes in the physiological responses during the transition from the 1st to the 24th contraction at 20, 40, and 60% MVC, with MAP as a covariate. Significant ‘F’ ratios were analyzed on a *post-hoc* basis using the Scheffe procedure for multiple comparisons. The Bonferroni correction procedure was applied to minimize the possibility of Type 1 error. Pearson correlations were used to examine relationships between force output and the RMS, cerebral and muscle hemodynamic variables at 20, 40, and 60% MVC. Values were considered significant at *P* < 0.05. All statistical analyses were performed using SPSS software (version 14.1).

## Results

### Force output and electromyographic responses at the three intensities

The mean values of the force output and RMS for the 24 intermittent isometric contractions of the biceps brachii at 20, 40, and 60% MVC are summarized in Table 1. The increase in force output across the three intensities was accompanied by significant increases in the RMS, both in absolute values and relative to the MVC. This was observed in all subjects across all exercise intensities, but with considerable inter-subject variability.

**Table 1:** **Electromyographic, cardiovascular and cerebrovascular responses at rest and during 24 intermittent isometric contractions of the biceps brachii at three intensities (Values are mean ± *SD*, *N* = 11)**.

**Variable**	**Rest**	**20% MVC^†^**	**40% MVC^†^**	**60% MVC^†^**
Force (N)	–	22.9 ± 7.8	45.2 ± 13.0^*^	64.9 ± 20.4^‡^
RMS, (mV)	–	0.18 ± 0.11	0.43 ± 0.28^*^	0.77 ± 0.41^‡^
RMS, %MVC	–	20.1 ± 11.5	37.9 ± 10.1^*^	68.7 ± 9.6^‡^
Mean MCAv, (cm.s^−1^)^a^	36.5 ± 7.9	49.8 ± 11.2	46.8 ± 10.3	49.3 ± 11.2
MAP (mmHg)	76.6 ± 9.9	86.6 ± 14.9	83.6 ± 19.3	91.8 ± 23.4
HR (bpm)	62.7 ± 6.5	75.2 ± 29.4	91.5 ± 23.1^*^	100.1 ± 23.0^‡^

a Mean MCAv is the average of the right and left values as there were no significant differences between the two sides.

† All exercise values shown are significantly different from the corresponding resting values “

* ” indicates significantly different from mean value at 20% MVC.

‡ indicates significantly different from mean value at 20 and 40% MVC.

### Cerebrovascular and cardiovascular responses at the three intensities

The mean values of the cerebrovascular and cardiovascular responses at the three intensities are summarized in Table 1. The right and left MCAv increased from the baseline value at all three intensities, with no differences observed among the intensities. Likewise, no differences were observed between the right and left MCAv during these contractions at all exercise intensities. MAP increased significantly above baseline, but there were no differences among the intensities. The increase in MAP was due to increases in both SBP and DBP during the biceps brachii contractions. Heart rate increased above the resting value and demonstrated a systematic increase across the three intensities.

### Cerebral and muscle hemodynamic responses at the three intensities

**Figure 1:**
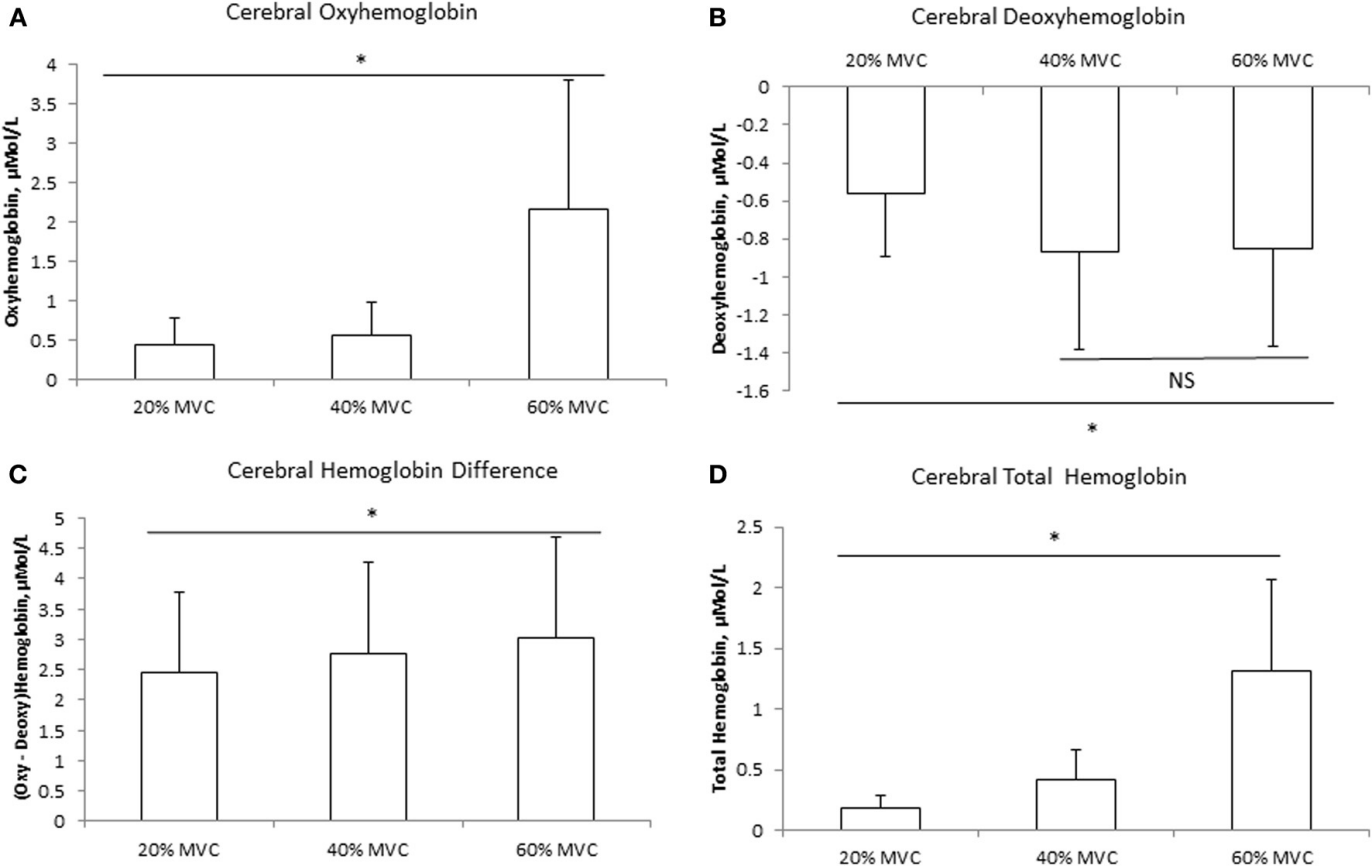
**Cerebral hemodynamic responses at three different intensities of intermittent isometric contractions of the biceps brachii**. ^*^Indicates *P* < 0.05.

**Figure 2:**
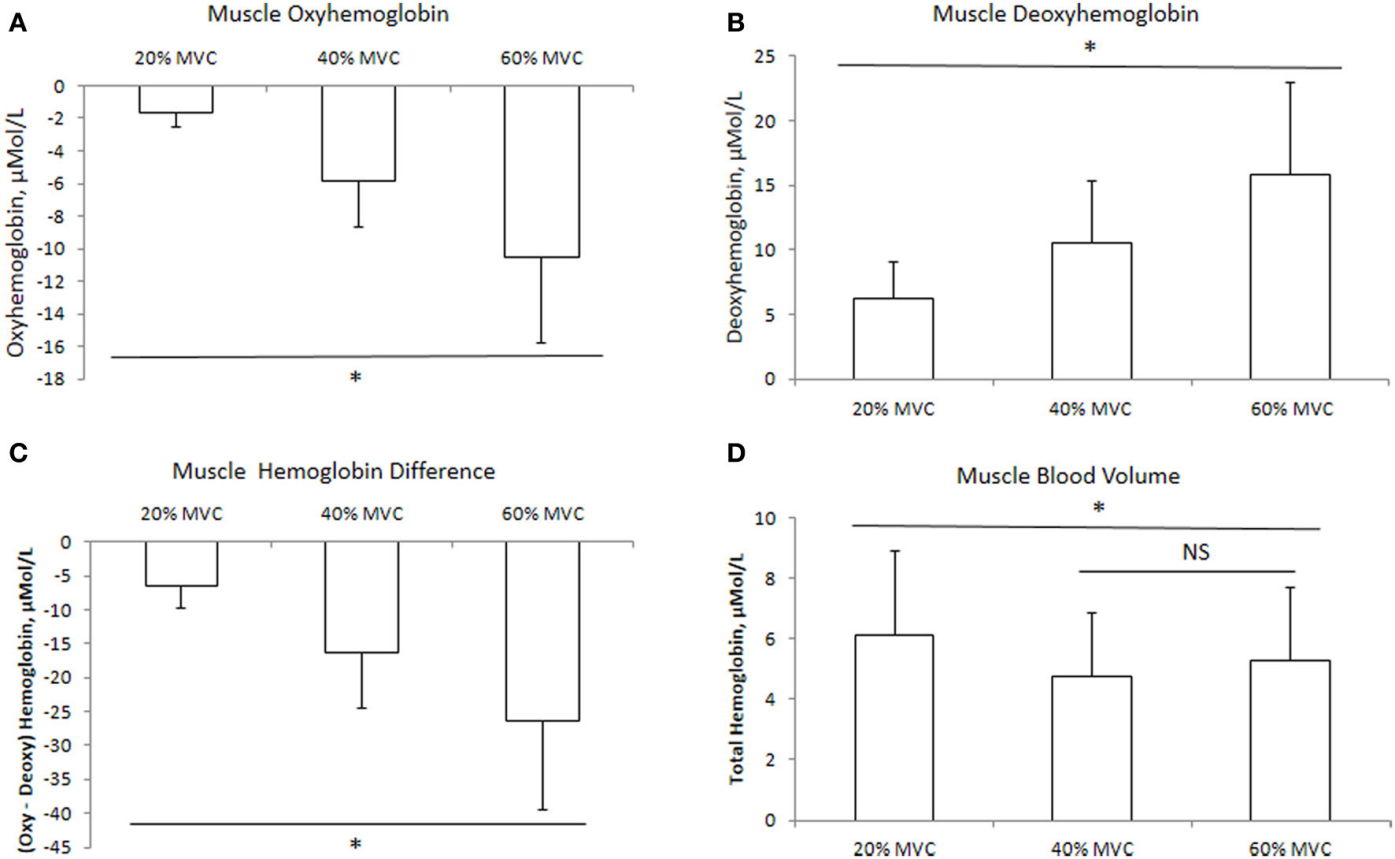
**Muscle hemodynamic responses at three different intensities of intermittent isometric contractions of the biceps brachii**. ^*^Indicates *P* < 0.05.

The mean delta values of the cerebral and muscle hemodynamic responses are illustrated in Figures 1, 2, respectively. Cerebral HbO_2_, Hb_diff_, and Hb_tot_ increased while HHb decreased with increasing intensity. There was no difference between 40 and 60% MVC for cerebral HHb. Muscle HbO_2_, Hb_tot_, and Hb_diff_ declined while HHb increased with increasing intensity. Hb_tot_ was higher at 20% MVC compared to 40 and 60% MVC but was not different between 40 and 60% MVC.

### Relationships between force output, electromyographic, and hemodynamic responses

Pearson correlations summarizing the relationships between force output, RMS, cerebral and muscle hemodynamic responses at the three intensities are presented in Table 2. The correlations between force output and RMS were significant at each intensity with a trend toward a stronger relationship at 20 and 40% MVC compared to 60% MVC. Force output was not significantly correlated with cerebral HbO_2_ and HHb at any intensity. However, it was significantly correlated with muscle HbO_2_ and was approaching significance with HHb at all three intensities. The correlations between RMS and cerebral as well as muscle HbO_2_ and HHb changes during the intermittent biceps brachii contractions at the three intensities are summarized in Table 3. RMS was not significantly correlated with the cerebral or muscle HbO_2_ and HHb at any of the intensities.

**Table 2:** **Pearson correlations and common variance (in parentheses) between force output and selected electromyographic, cerebral, and muscle hemodynamic variables during intermittent contractions of the biceps brachii at three intensities (*N* = 11, critical ‘*r*’ value = 0.58)**.

**Variable**	**Pearson r (% *r*^2^) between force output and variables at three intensities**		
	**20% MVC**	**40% MVC**	**60% MVC**
RMS	0.73^*^ (53.6%)	0.71^*^ (50.2%)	0.59^*^ (34.8%)
Delta cerebral HbO_2_	0.46 (21.4%)	0.19 (3.1%)	0.24 (5.6%)
Delta cerebral HHb	0.35 (12.2%)	0.33 (10.9%)	0.04 (1.8%)
Delta muscle HbO_2_	0.64^*^ (41.0%)	0.62^*^ (38.4%)	0.63^*^ (39.8%)
Delta muscle HHb	0.54 (29.5%)	0.57 (32.5%)	0.51 (26.0%)

* Correlation significant at P < 0.05. Note: force output was significantly correlated with RMS at 20 and 40% MVC but not at 60% MVC. Force output was significantly correlated with muscle HbO_2_ but not cerebral HbO_2_, cerebral HHb and muscle HHb.

**Table 3:** **Pearson correlations between electromyographic and cerebral/muscle hemodynamic responses during intermittent contractions of the biceps brachii at three intensities (*N* = 11, critical ‘*r*’ value = 0.58)**.

**Intensity**	**Cerebral HbO_2_**	**Cerebral HHb**	**Muscle HbO_2_**	**Muscle HHb**
RMS at 20% MVC	0.44	0.03	−0.16	0.25
RMS at 40% MVC	0.51	−0.05	−0.19	0.09
RMS at 60% MVC	0.49	−0.11	−0.25	0.16

None of the correlations were significant at P < 0.05.

### Transitional changes in force output, electromyographic, and hemodynamic responses at the three intensities

**Figure 3:**
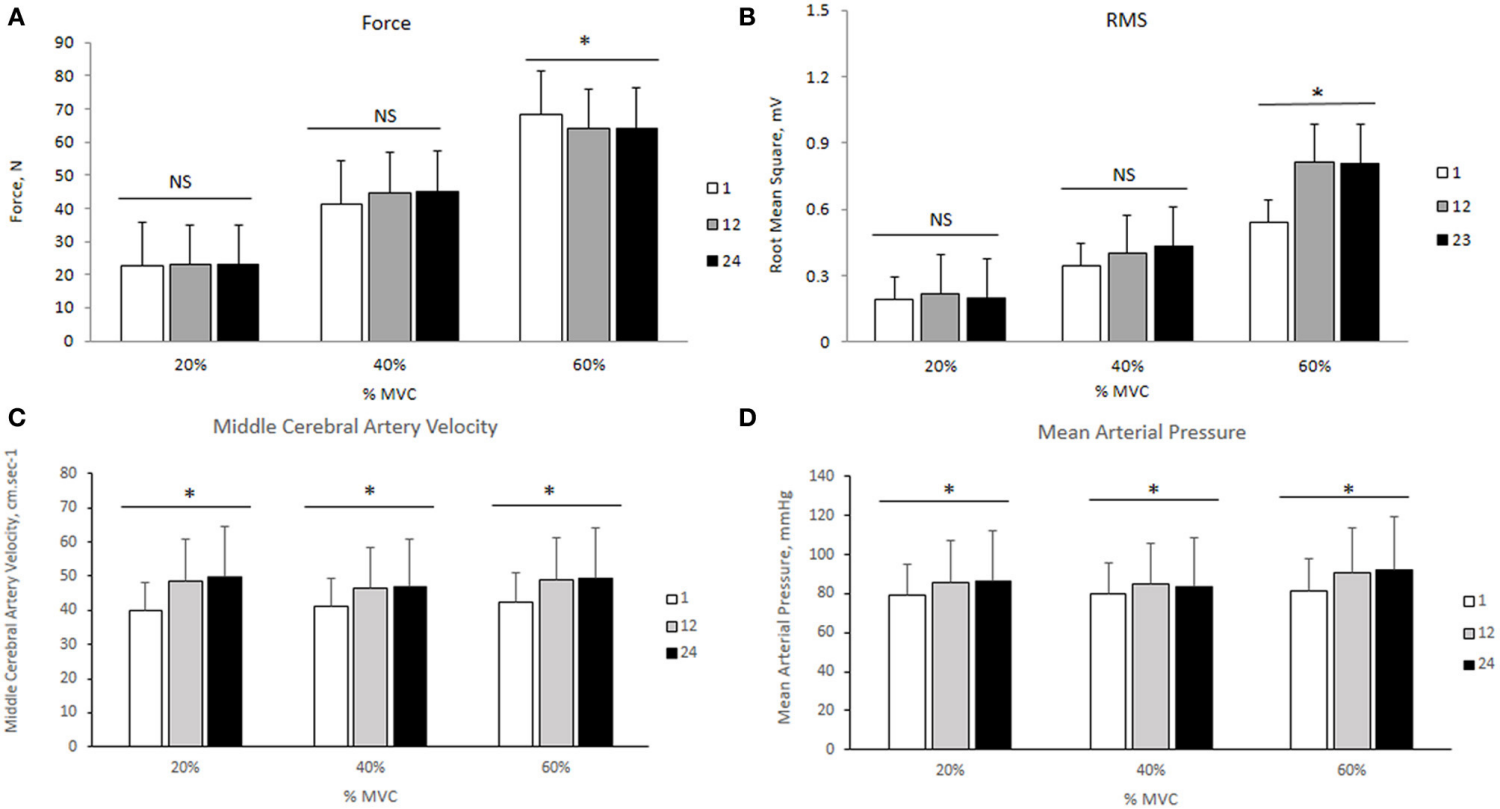
**Force output, root mean square, middle cerebral artery velocity, and mean arterial pressure during 24 intermittent isometric contractions of the biceps brachii at three different intensities**. ^*^Indicates *P* < 0.05.

**Figure 4:**
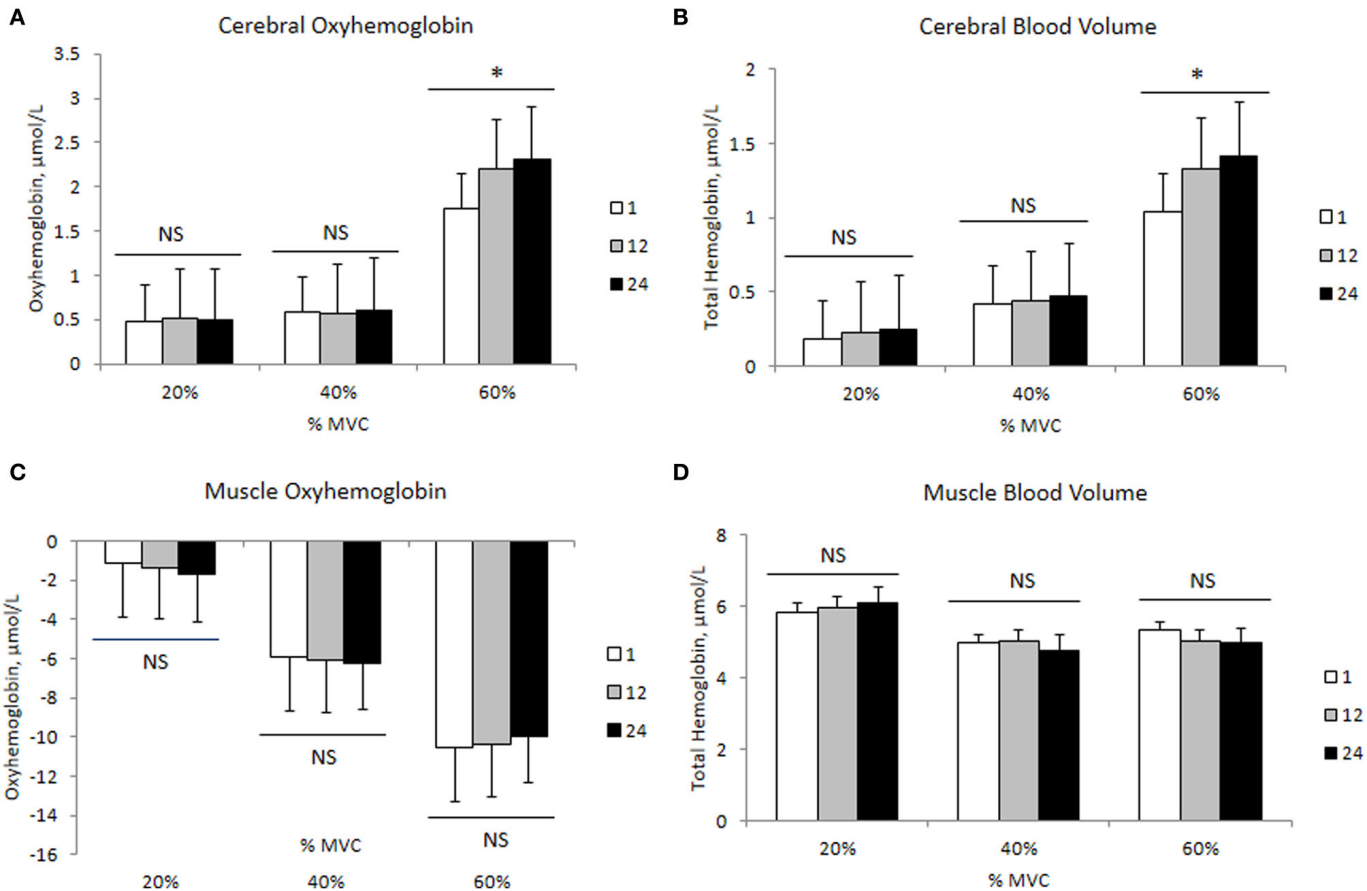
**Cerebral and muscle hemodynamic changes during 24 intermittent isometric contractions of the biceps brachii at three different intensities**. ^*^Indicates *P* < 0.05.

**Figure 5:**
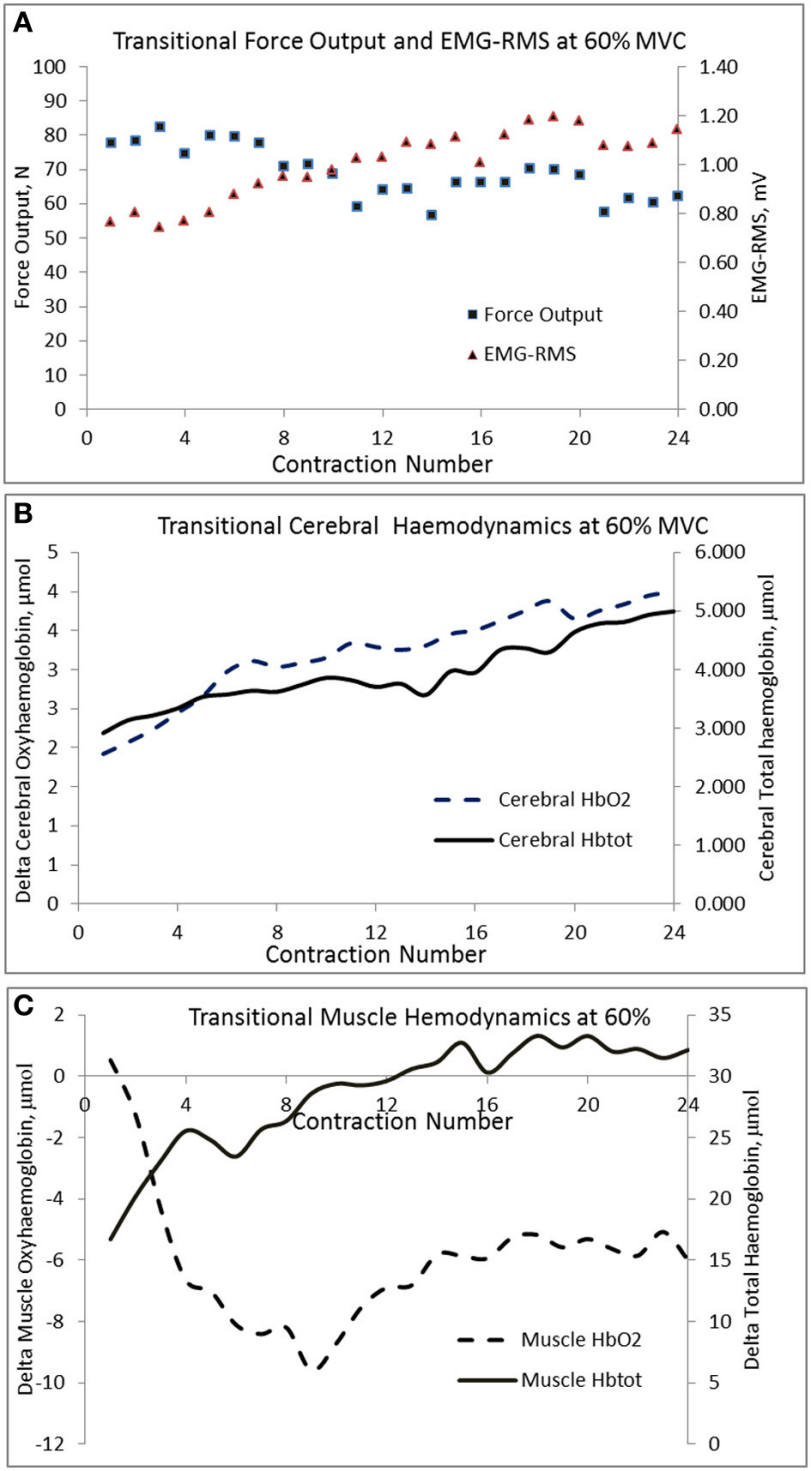
**Transitional changes in force output, cerebral, and muscle hemodynamic responses during the 24 contractions of the biceps brachii at 60% MVC in a representative subject**.

The changes in mean force output and RMS during the transition from the 1st to the 24th contraction at each intensity are illustrated in Figures 3A,B, respectively. At 20 and 40% MVC, there were no changes in the force output and RMS from the 1st to the 24th contraction. However, at 60% MVC force output declined by 8% from the 1st to the 12th and 24th contractions and was unchanged between the 12th and 24th contractions. This decline in force output was accompanied by an increase in RMS. Increases in MCAv and MAP were observed between the 1st to the 12th contraction, but not from the 12th to the 24th contraction at each intensity (Figures 3C,D, respectively). The changes in cerebral and muscle HbO_2_ and Hb_tot_ are illustrated in Figures 4A–D, respectively. Cerebral HbO_2_ and Hb_tot_ increased progressively from the 1st to the 24th contraction with no sign of leveling off. In contrast, muscle HbO_2_ and Hb_tot_ demonstrated a decline from the 1st to the 12th contraction and a plateau during the remaining 12 contractions. A representative trend is available in Figures 5A–C.

## Discussion

This study used a multi-modal, non-invasive approach to simultaneously evaluate the electromyographic, cerebrovascular and hemodynamic responses during intermittent, isometric elbow flexor contractions at 20, 40, and 60% MVC. Before discussing the physiological implications of our findings, we first address some of the methodological considerations of these non-invasive techniques.

### Methodological considerations

Electromyography in conjunction with NIRS is used to non-invasively evaluate cerebral and muscle activation during exercise involving large muscle volume. Alterations in RMS are thought to reflect changes in central activation influencing force output during various types of muscle contractions. However, EMG signals do not allow us to differentiate between the supraspinal (cortical) and spinal contributions to muscle activation (Burnley et al., 1985; Millet et al., 2011). Meanwhile, the validity of cerebral NIRS for assessing cortical oxygenation status has been compared with MRI measurements (Huppert et al., 2006; Schroeter et al., 2006). An increase in cerebral HbO_2_ with a concomitant decline in HHb reflects enhanced neuronal activation via neurovascular coupling (Rasmussen et al., 2010). Such hemodynamic responses are accompanied by enhanced oxygen, glucose and lactate utilization, which reflects increased metabolic needs of the activated neurons (Dalsgaard and Secher, 2007). Given the close relationship between measurements from the left prefrontal lobe and the motor cortex, prefrontal lobe changes are used as a surrogate for alterations in the motor cortex (Ainslie et al., 2007; Subudhi et al., 2009). Nevertheless, our cortical tissue measurements should be interpreted with caution as prefrontal NIRS signals can be influenced by changes in scalp blood flow (Payne et al., 2011). While the decline in HbO_2_ accompanied by a plateau or increase in HHb indicates enhanced oxidative metabolism of contracting fibers (Mancini et al., 1994; Bhambhani, 2012), the muscle NIRS signals are directly influenced by adipose tissue thickness (van Beekvelt et al., 2001). However, representative measurements are obtained in subjects with a BMI less than 32 (McCully and Hamaoka, 2000); a criterion met by all the subjects in this study. Transcranial Doppler ultrasound measures blood flow velocity *per se*, rather than flow. While the MCAv changes during dynamic incremental exercise have been shown to reflect changes in internal carotid blood flow (Sato et al., 2011), we cannot exclude the possibility that the MCA diameter increased during isometric contraction, which would lead to an underestimation of CBF changes. Finally, this study only assessed the RMS and hemodynamic responses of the biceps brachii. It should be recognized that the brachialis and brachioradialis muscles are also activated during sustained elbow flexion, with differential load sharing patterns during submaximal isometric contractions (Bouillard et al., 2012). If such a similar load-sharing pattern occurred during the intermittent isometric contractions in this study, the muscle hemodynamic responses may have differed among these three muscles and altered the interpretation of these results.

### Electromyographic responses at the three intensities

The increased force output of the biceps brachii during the intermittent isometric contractions at the three intensities was accompanied by an increase in biceps brachii RMS expressed in absolute values or relative to the MVC (Table 1). Although there was a tendency for RMS to increase disproportionately at 60% MVC compared to 20 and 40% MVC, these differences were not statistically significant. This was most likely due to the fact that we did not exercise the subjects to voluntary fatigue, but terminated the test after a finite number of muscle contractions (Felici et al., 2009) in our protocol. As well, the contractions were intermittent in nature as supposed to being sustained, which would have enabled some recovery in the neuromuscular responses between the successive contractions. The increase in RMS implies enhanced motor unit firing frequency and/or greater motor unit recruitment to generate the desired force output at the higher exercise intensities (Felici et al., 2009; Neyroud et al., 2013). In accordance with the Henneman size principle (Henneman et al., 1965), it is likely that there was a systematic progression in the recruitment starting with the Type 1, low activation threshold (slow twitch) motor units, followed by inclusion of the fast-fatigable Type II motor units, in order to develop the desired force at the higher intensities.

### Cerebrovascular responses at the three intensities

Mean MCAv increased from the resting baseline value at all three intensities during the unilateral intermittent contractions of the biceps brachii (Table 1). This observation is consistent with previous reports of increased MCAv during rhythmic and sustained hand grip contractions at submaximal and maximal intensities (Giller et al., 2000; Zebrowska et al., 2013)—presumably due to increased central command (Vianna et al., 2009). However, since there is considerable heterogeneity in MCAv responses during maximal intermittent isometric exercise between individuals, with some subjects demonstrating an increase and others showing a decrease, these findings should be viewed with caution (Giller et al., 2000).

One of the novel findings of the present study was that the rise in MCAv during isometric contraction was the same between different contraction intensities, with no difference between the hemispheres, despite an increase in heart rate (Table 1). We attribute this lack of difference in MCAv rise between contraction intensities to the: (1) intermittent nature of the contractions (i.e., 2.5 s contraction followed by 2.5 s relaxation); and (2) avoidance of the Valsalva maneuver by exhaling during the contractions. Both these factors would have attenuated the expected blood pressure rise and associated increase in MCAv during the contraction period (Haykowsky et al., 2003; Zebrowska et al., 2013). In support, we found no difference in MAP among the three intensities (Table 1). We found the increase in prefrontal Hb_tot_ was greater at higher exercise intensity (Figure 2D). Since this occurred in the absence of a greater increase in MCAv (Table 1), we speculate that this increase in Hb_tot_ was likely due to a redistribution of the CBF increase during isometric elbow flexion. Taken together, these findings indicate that, when controlled for MAP, global CBF increases by the same extent during low, moderate and heavy intermittent isometric contractions.

### Cerebral and muscle hemodynamic responses at the three intensities

Consistent with our hypothesis, mean cerebral HbO_2_ increased while HHb decreased progressively with increasing exercise intensity (Figures 1A,B), independent of changes in MAP. These trends are consistent with previous measurements during intermittent isometric elbow flexion performed at 30 and 100% MVC (Muthalib et al., 2012) and during repeated maximal hand grip contractions (Bhambhani et al., 2006). The increases in cerebral HbO_2_ and Hb_diff_, coupled with the concomitant decrease in HHb suggest enhanced neuronal activation (Dalsgaard and Secher, 2007; Ogoh and Ainslie, 2009). This supports our EMG observations of increased RMS (as an index of neural drive) with increasing intensity from 20 to 60% MVC. Therefore, our findings indicate that the increased motor-neuronal activation and associated increased motor recruitment with progressively higher exercise intensities can be observed with our cerebral NIRS measurements.

Using positron emission tomography, Korotkov et al. (2005) reported increased regional CBF during sustained isometric elbow flexion at 30–50% MVC. Specifically, they found blood flow to the primary and secondary somatosensory areas, the somatosensory association area, and the temporal area contralateral to the muscle, to increase with increasing intensity and duration of the fatiguing contractions. Furthermore, they found these regional CBF increases were associated with enhanced cortical activation, which spread to several cortical areas and reflected the changes in both excitatory and inhibitory cortical circuits. In the present study, we found increases in cerebral HbO_2_ and Hb_tot_ were disproportionately greater, relative to force output, at 60% MVC (Figure 1D). We speculate this disproportionate increased prefrontal activation and blood flow might be due to reduced efficiency during high intensity contractions. In support, visual observation indicated that the subjects began to recruit accessory muscle, such as the trunk and shoulder muscles, to maintain the target force for this intensity. This would lead to additional neuronal activation, which may account for the exaggerated increase in cerebral HbO_2_ observed at 60% MVC.

In agreement with previous research (Felici et al., 2009; Muthalib et al., 2010a,b), muscle HbO_2_ decreased with increasing intensity while muscle HHb increased (Figures 3A,B). Praagman et al. (2003) reported a significant relationship between force output and muscle oxygenation during sustained isometric elbow flexion up to 70% MVC. Similarly, we found a strong relationship between these two variables between 20 and 60% MVC (Table 2). We interpret this as increased oxygen extraction in order to meet the increased aerobic ATP production during effort and associated increase in motor unit recruitment. It should be noted that these trends in HbO_2_ and HHb were evident during the entire sequence of contractions (from 1 to 24), even though they were intermittent in nature. Perhaps surprisingly, we observed no reoxygenation during the 2.5 s relaxation periods between the contractions. We speculate that this might reflect the restoration of oxymyoglobin and phosphocreatine in the muscle (Kime et al., 2003). During exercise, the increase in Hb_tot_ was higher at 20% MVC compared to 40 and 60% MVC (Figure 2D). We attribute this to a lower intramuscular pressure during the contraction and relaxation phase at 20% MVC, which allows for better reperfusion of the tissue, thereby increasing the muscle blood measured by NIRS. Since the Hb_tot_ increase was not different between 40 and 60% MVC, we speculate that a threshold intramuscular pressure for blood was attained above 20% MVC. This is consistent with a previous study (Zwarts and Arendt-Nielsen, 1988) which found muscle blood flow to be reduced during sustained elbow flexion at 50–60% MVC, but not at 10% MVC.

### Relationship between neuromuscular and hemodynamic responses

In contrast to our hypothesis, we found no correlation between the RMS with either the cerebral or muscle hemodynamic responses (HbO_2_ and HHb) at any of the exercise intensities (Table 3). Our findings contrast those by Felici et al. (2009) which reported a significant relationship between RMS and muscle tissue oxygen saturation (ratio between HbO_2_ and Hb_tot_) during sustained elbow flexion at 20–80% MVC. From this, they concluded that these two techniques provided complementary information pertaining to motor unit recruitment and muscle oxidative metabolism. The lack of a significant relationship between EMG and NIRS results in the present study could be due to the large inter-individual variability of these responses and the limited number of participants.

### Transitional changes in force output, electromyographic, and hemodynamic responses at the three intensities

We observed a significant decline in force output between the 1st to the 24th intermittent isometric contraction at 60% MVC while no such decline was seen at 20 and 40% MVC. Since RMS, cerebral HbO_2_ and Hb_tot_ showed a consistent increase during this transition, while a plateau was observed in both MCAv and MAP, it seems unlikely that this decline in force output was due to a reduction in neuronal activation. Instead, we believe the increase in RMS, in the face of declining force output, may be attributed to: (1) greater recruitment of fast but more fatigable motor units (Type II) (Gandevia, 2001; Enoka and Duchateau, 2008), (2) increased synchronization of motor units (Gandevia, 2001; Enoka and Duchateau, 2008), and (3) slowing of muscle fiber action potential conduction velocity in order to meet the force demands (Lindstrom et al., 1970). Neyroud et al. (2012) proposed that during a sustained isometric contraction of the biceps brachii at 50% MVC, the development of peripheral muscle fatigue was due to reduced muscle blood flow and oxygen availability. The data from the present study appears to give support to this hypothesis. Recently it was suggested that during intermittent isometric knee extension to exhaustion a critical threshold exists, above which the decline in force output was primarily induced by peripheral metabolic alterations rather than central neuronal activation (Burnley et al., 1985). Our results support the existence of such a threshold during intermittent elbow flexion, a hypothesis which should be tested further. Our findings are of potential use in developing scientifically sound exercise training regimens to enhance muscle strength and endurance in order to delay fatigue.

## Conclusions

Intermittent isometric elbow flexion performed at 20, 40, and 60% of MVC for 2 min (24 contractions) elicited significant increases in RMS and prefrontal cortex HbO_2_ with concomitant decreases in HHb. These findings are indicative of enhanced neuronal activation with increasing exercise intensity. Meanwhile, the progressive muscle HbO_2_ declined during increasing effort, coupled with elevations in HHb and Hbtot, implying greater oxygen utilization by the contracting muscle fibers. Finally, the development of muscle fatigue during intermittent isometric contraction at 60% MVC, accompanied by systematic increases in RMS, as well as cerebral HbO_2_ and Hb_tot_ with a leveling off in muscle HbO_2_ and Hb_tot_ suggests that muscle fatigue at this intensity was partly due to reduced peripheral oxygen availability rather than impaired central neuronal activation.

## Author contributions

All five co-authors actively participated in: (1) designing this project, (2) conducting the pilot studies, and (3) data collection. The experimental data were examined and evaluated by Yagesh Bhambhani and Jui-Lin Fan. The statistical analyses were completed by Yagesh Bhambhani. The first draft of the manuscript was written by Yagesh Bhambhani and reviewed by each co-author. The comments of the co-authors were incorporated into the manuscript by Yagesh Bhambhani. The final submission to Frontiers in Physiology was completed by Yagesh Bhambhani.

### Conflict of interest statement

The authors declare that the research was conducted in the absence of any commercial or financial relationships that could be construed as a potential conflict of interest.
